# A comprehensive study of medically serious suicide attempts in France: incidence and associated factors

**DOI:** 10.1017/S2045796022000774

**Published:** 2023-01-10

**Authors:** J. Corbé, C. Montout, A. Fares, I. Belhadj, T. Boudemaghe, T. Mura, J. Lopez-Castroman

**Affiliations:** 1 Department of Adult Psychiatry, Nimes University Hospital, Nimes, France; 2 Department of Biostatistics, CHU Nimes, Nimes University Hospital, Nimes, France; 3 Institut de Génomique Fonctionnelle, Université de Montpellier, CNRS, INSERM, Montpellier, France; 4 SIMMER, Nimes University Hospital, Nimes, France; 5 CIBERSAM, Madrid, Spain

**Keywords:** Mental health, public health, self-harm, social epidemiology

## Abstract

**Aims:**

People who make medically serious suicide attempts (MSSAs) share a number of features with those who die by suicide, and are at a high risk of suicide themselves. Studies to date have mostly focused on clinical samples of MSSAs. An epidemiological examination at a national level can help to identify risk profiles and pathways of care in this population.

**Methods:**

We explored the French nationwide hospital discharge database (Programme de Médicalisation des Systèmes d'Information, PMSI) to identify any MSSA taking place between 2012 and 2019. Relevant demographic and medical information was collected about the first MSSA of each attempter. Data from 2010 and 2011 were used to verify the absence of prior attempts.

**Results:**

First occurrences of MSSAs amounted to 81 959 cases over 8 years, with a mean age of 45.8 years, and 53.6% women. Incidence was higher in women (18.1 *v*. 17.3 per 1 00 000). The most common suicide method was deliberate self-poisoning (64.9% of cases). In comparison, violent methods associated higher mortality and comorbidity and were more frequent in men. The most common mental disorders were mood disorders (55.6%) and substance use disorders (46.2%). A minority of MSSA survivors were hospitalised in psychiatry (32.5%), mostly women.

**Conclusions:**

MSSAs are frequent and easy to identify. There is a need to reinforce the continuity of psychiatric care for this population given the high risk of subsequent suicide, and the low rates of psychiatric hospitalisation after an MSSA even if violent methods are used. Specific care targeting this population could reduce treatment gaps.

## Introduction

It is estimated that for every suicide death there are about 10–30, depending on the country, non-lethal suicide attempts (SAs) (Mościcki *et al*., 1988; Kessler *et al*., 2005; Han *et al*., 2016; Bachmann, 2018). Having attempted suicide constitutes the best predictor of suicide since about 40% of victims of suicide have records of previous attempts (Hawton *et al*., 2005; Hawton and van Heeringen, 2009), multiplying the risk of suicide in the year following an attempt by 49 (Hawton *et al*., 2015). However, not all SAs convey the same risk.

A medically serious suicide attempt (MSSA) has been defined as an SA that would have been fatal without access to emergency care and that subsequently required hospitalisation for more than 24 h in an intensive care unit (ICU), or surgery under general anaesthesia, regardless of the violence of the act (Levi-Belz and Beautrais, 2016). MSSAs constitute a subsample of all SAs, and thus imply a potentially self-injurious behaviour, associated with at least some intent to die (Posner *et al*., 2007). The main difference between MSSAs and other SAs (low-lethality SAs) is the medical lethality of the attempt and, correspondingly, the level of subsequent medical care needed to keep the person alive. Establishing a cut-off based on the medical lethality of the attempt has been proposed as the best method to differentiate serious SAs, i.e. those ‘that would have been lethal had it not been for the provision of rapid and effective emergency treatment’ (Gvion and Levi-Belz, 2018). Compared to low-lethality SAs, individuals who make MSSAs are phenotypically closer to those who die by suicide (Mościcki *et al*., 1988; Beautrais, 2003a; Gvion and Levi-Belz, 2018). They are older, more likely to have prior records of SAs and report higher lethality in previous SAs compared to attempters that never made an MSSA (Giner *et al*., 2014). Mental disorders and suicide methods in MSSAs are also half-way between suicides and SAs. Many studies report that self-poisoning is majoritarian in MSSA samples (57–79%) but violent methods such as hanging are overrepresented (10–17%) in comparison to SAs with less serious consequences (Beautrais, 2003b; Horesh *et al*., 2012; Sun *et al*., 2015; Kim *et al*., 2020). Bipolar disorder, substance misuse and eating disorder are also overrepresented in MSSA samples (Giner *et al*., 2014). Conversely, the diagnoses of non-affective psychosis seem to be much more common in samples of suicide victims than in MSSA samples (OR = 8.5) (Beautrais, 2001).

Among suicide attempters, making an MSSA more than doubles the risk of completing suicide in the short term. One in 25 individuals (4%) die by suicide in the 18 months that follow an MSSA (Beautrais, 2004), but only 1.8% in the year after an SA (Probert-Lindström *et al*., 2020). This is also true in the medium term (over 5 years): 5.3–7% of MSSA attempters die by suicide (Beautrais, 2003a, 2004) compared to 3.8% if we consider all the attempters (Probert-Lindström *et al*., 2020). The overall suicide mortality after an MSSA is multiplied by 5 compared to the general population (Beautrais, 2003a). Importantly, the direct assessment of individuals who made an MSSA, and could have died by suicide, provides proxy information about mental disorders, cognitive processes or psychological traits in suicide deaths (Clark and Horton-Deutsch, 1992; Hawton *et al*., 1998; Hawton, 2001).

We have scarce epidemiological information about MSSAs. Only some clinical studies investigated the somatic and psychiatric comorbidity associated to them. Here, we aimed to describe from a demographic and clinical standpoint the population that makes a first MSSA and what use they make of hospital care at a national level. We will also calculate the incidence of MSSAs. To our knowledge, only one study has done it before but it included suicide deaths before arrival at the hospital (Sun *et al*., 2015). Indeed, most previous studies were conducted on clinical samples of no more than 1500 individuals (Beautrais *et al*., 1996; Beautrais, 2001, 2004; Kim *et al*., 2020). Our database provides exhaustive information on the entire French population admitted to hospitals between 2012 and 2019. Because of the small amount of data available on this topic and the high-risk profile of MSSA survivors, we believe that this study will provide useful information to caregivers in the management of suicidal behaviour.

## Methods

### Study design and data source

We conducted a nationwide observational study in French hospitals using the French nationwide hospital discharge database (*Programme de Médicalisation des Systèmes d'Information*, PMSI). The PMSI database contains synthetised and anonymised data about all the units of medical establishments providing acute care in France, which encompasses medicine, surgery and obstetrics (*Medecine, chirurgie, obstétrique*, MCO). Conventional psychiatric units are not included in the MCO sector. PMSI data are prospectively collected by all public and private hospitals in a standardised way for care reimbursement purposes. Every discharge summary contains sociodemographic information about the patient, medical information about the hospital stay and data related to the trajectory of the patient. Medical information is coded with the Tenth revision of the International Classification of Diseases (ICD-10).

National PMSI data are anonymised. Access to these data is authorised for research purposes and does not require the individual information and written consent of the patients. Approval of the National Data Protection Commission (*Commission Nationale Informatique et Liberté*, CNIL) was obtained for this study and data were handled and analysed on the secured electronic platform of the National Agency for the Information on Hospitalisations (*Agence Technique de l'Information sur l'Hospitalisation*, ATIH).

### Study population

All the inpatient admissions related to a first episode of MSSA in the acute care sector of French hospitals between 2010 and 2019 were considered for the present study. A hospital stay for an MSSA implied: (1) the presence of an intentional self-harm ICD-10 code X60–X84 in the discharge summary; (2) the admission to an ICU for somatic conditions, including critical care units; and (3) the absence of previous hospitalisations caused by an MSSA for at least the two preceding years. The 2-year period comprises the years 2010 and 2011 for MSSAs that took place in the year 2012, or the longest period available since 2010 for MSSA that took place after 2012. To ensure that we were studying a homogenous sample, only first admissions fulfilling the criteria for MSSA were selected.

### Study variables

1. Patients' demographics (sex and age), and comorbidities according to the Charlson index algorithm. The Charlson Comorbidity Index categorises a range of non-psychiatric comorbidities of patients based on the ICD to predict short-term mortality. The 1-year mortality rates for the different scores are: ‘0’, 12%; ‘l–2’, 26%; ‘3–4’, 52%; and ‘ >5’, 85% (Charlson *et al*., 1987).
2. Medical variables: primary/main and secondary diagnoses coded with the ICD-10 related to organic and psychiatric comorbidities, and method of the SA. To summarise diagnostic data, we used ICD-10 chapter codes for mental disorders (F0 to F9). Three specific diagnoses were analysed separately because of their strong association with suicide risk: bipolar disorder (F30–F31), alcohol use disorder (AUD) (F10) and schizophrenia (F20).
3. Variables related to the patients' intra and inter-hospital trajectory: lengths of stay, admission in ICU, origin of referral and destination, vital status at discharge.
4. Admission in a psychiatric hospital within 3 days of discharge from the MCO units.

### Statistical analysis

We performed descriptive analyses to characterise the sample. Qualitative variables were reported with numbers and percentages; quantitative ones, with means and standard deviations (SD). Comparison between groups were carried out using the Pearson *χ*^2^ tests for qualitative variables (or Fischer exact tests for small groups as appropriate), and using the Student *t*-test for quantitative variables.

To estimate the incidence rates of first MSSA in the French general population, we used as numerator the exhaustive number of first MSSAs in France by age, sex and year (estimated by our whole population study), and as denominator the size of the French population by age, sex and year estimated by the French ‘National Institute of Statistics and Economic Studies’ (INSEE). These population sizes are based on an annual national census and are directly available on the Institute's website (Papon & Beaumel, 2020).

Analyses were performed with a bilateral *α* level of 0.05 using the SAS Enterprise guide 7.15 (SAS Institute, Cary, North Carolina, USA).

## Results

**Fig. 1.**
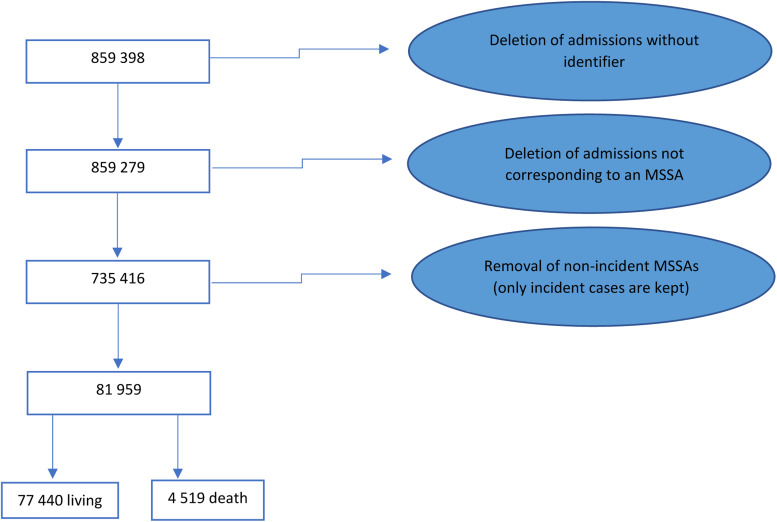
Data flowchart, selection of incident MSSA.

A total of 7 35 416 MSSAs were found between 1 January 2012 and 31 December 2019. Among them, 81 959 first-time MSSAs were identified and analysed (see data flowchart in Fig. 1). As we consider only the first MSSAs for the present study, one patient corresponds to one discharge summary.

### Sample description by sex

**Fig. 2.**
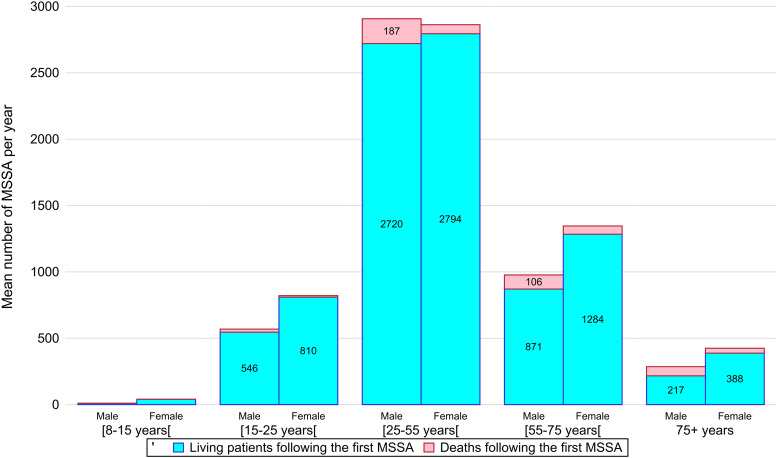
Stacked column chart representing the distribution by age group and gender of the average number of first medically serious suicide attempts (MSSAs) during the study period. Deaths following the first MSSA are shown on top of the columns.

Women represent about a half of MSSA cases (*N* = 43 953, 53.62%; *p* < 0.0001), and a larger majority in extreme age groups (under 25 or over 55 years) (Table 1, Fig. 2). In the age group between 25 and 55 years, which counts with the largest caseload (*N* = 46 150, 56.3%), men are overrepresented (*N* = 23 258, 50.4%). MSSAs before 15 are rare in both sexes (*N* = 412, 0.5%).

**Table 1.** Demographic and clinical features of all MSSA patients according to their sex

	Male *N* = 38 006 *N* (%)	Female *N* = 43 953 *N* (%)	Total *N* = 81 959 *N* (%)	*p*-Value^a^
Age (mean ± s.d.)	45.04 ± 16.81	46.45 ± 18.31	45.8 ± 17.64	**<0.0001**
Age in five classes				**<0.0001**
[8 years; 15 years]	89 (0.2%)	323 (0.7%)	412 (0.5%)	
[15 years; 25 years]	4550 (12%)	6570 (14.9%)	11 120 (13.6%)	
[25 years; 55 years]	23 258 (61.2%)	22 892 (52.1%)	46 150 (56.3%)	
[55 years; 75 years]	7817 (20.6%)	10 766 (24.5%)	18 583 (22.7%)	
75+ years	2292 (6%)	3402 (7.7%)	5694 (6.9%)	
Drug poisoning (X60–X64)	21 472 (56.5%)	31 695 (72.1%)	53 167 (64.9%)	**<0.0001**
Death because of the attempt	3090 (8.1%)	1429 (3.3%)	4519 (5.5%)	**<0.0001**
Admission				
From the emergency room	25 073 (66%)	29 185 (66.4%)	54 258 (66.2%)	0.31
From a psychiatric hospital	134 (0.4%)	164 (0.4%)	298 (0.4%)	
From home	11 731 (30.9%)	13 443 (30.6%)	25 174 (30.7%)	
Other	1048 (2.8%)	1147 (2.6%)	2195 (2.7%)	
Discharge				
Home	17 638 (46.4%)	20 014 (45.5%)	37 652 (45.9%)	**<0.0001**
To a psychiatric hospital	10 456 (27.5%)	14 721 (33.5%)	25 177 (30.7%)	
Other	6822 (17.9%)	7789 (17.7%)	14 611 (17.8%)	
Days in intensive care (mean ± s.d.)	5.55 ± 10.12	4.4 ± 7.39	4.93 ± 8.78	**<0.0001**
Charlson score				
0 (1-year mortality = 12%)	33 553 (88.3%)	39 839 (90.6%)	73 392 (89.5%)	**<0.0001**
1–2 (1-year mortality = 26%)	2897 (7.6%)	3041 (6.9%)	5938 (7.2%)	
3–4 (1-year mortality = 52%)	1035 (2.7%)	652 (1.5%)	1687 (2.1%)	
5+ (1-year mortality = 85%)	521 (1.4%)	421 (1%)	942 (1.1%)	
ICD 10 codes for mental and behavioural disorders^b^				
F01–F09	1212 (4.4%)	1187 (3.7%)	2399 (4.1%)	**<0.0001**
F10–F19	15 423 (56.3%)	11 923 (37.5%)	27 346 (46.2%)	**<0.0001**
F20–F29	2698 (9.8%)	1814 (5.7%)	4512 (7.6%)	**<0.0001**
F30–F39	12 548 (45.8%)	20 339 (64%)	32 887 (55.6%)	**<0.0001**
F40–F48	2989 (10.9%)	4942 (15.6%)	7931 (13.4%)	**<0.0001**
F50–F59	101 (0.4%)	573 (1.8%)	674 (1.1%)	**<0.0001**
F60–F69	908 (3.3%)	1139 (3.6%)	2047 (3.5%)	0.07
F70–F79	79 (0.3%)	51 (0.2%)	130 (0.2%)	**0.0009**
F80–F89	37 (0.1%)	22 (0.1%)	59 (0.1%)	0.01
F90–F98	344 (1.3%)	497 (1.6%)	841 (1.4%)	**0.002**
F99	293 (1.1%)	422 (1.3%)	715 (1.2%)	**0.004**
Alcohol use disorder	11 733 (42.8%)	8593 (27.1%)	20 326 (34.3%)	**<0.0001**
Bipolar disorder	1407 (5.1%)	2843 (8.9%)	4250 (7.2%)	**<0.0001**
Schizophrenia	1504 (5.5%)	681 (2.1%)	2185 (3.7%)	**<0.0001**
Psychiatric admission in the 3 days following discharge	12 309 (32.4%)	16 912 (38.5%)	29 221 (35.7%)	**<0.0001**

a *p*-Value for comparison between males and females, we used the Pearson *χ*^2^ tests for qualitative variables and the Student *t*-test for quantitative variables.

b F00–F09: organic, including symptomatic, mental disorders; F10–F19: mental and behavioural disorders due to psychoactive substance use; F20–F29: schizophrenia, schizotypal and delusional disorders; F30–F39: mood (affective) disorders; F40–F48: neurotic, stress-related and somatoform disorders; F50–F59: behavioural syndromes associated with physiological disturbances and physical factors; F60–F69: disorders of adult personality and behaviour; F70–F79: mental retardation; F80–F89: disorders of psychological development; F90–F98: behavioural and emotional disorders with onset usually occurring in childhood and adolescence; F99: unspecified mental disorder.

Bold text indicates *p*-values ≤ 0.5.

Deliberate self-poisoning (DSP) by drugs is the most common method of SA in MSSA (64.9% of the cases), especially among women (72.1% compared to 56.5% among men). Men are more likely to use violent methods such as hanging (7.9 *v*. 1.9% in women) or firearms (3.1 *v*. 0.2%). Men are also more likely to die after their first MSSA (8.1 *v*. 3.3%).

Concerning mental disorders, the most frequent diagnoses are mood disorders (55.6%), including bipolar disorder (7.2%), and substance use disorders (SUDs) (46.2%), including alcohol dependence (34.3%). Compared to women, men seem to suffer significantly more from SUDs (56.3 *v*. 37.5%), particularly alcohol dependence (42.8 *v*. 27.1%), and schizophrenia (5.5 *v*. 2.1%). Conversely, women are more concerned by mood disorders (64 *v.* 45.8%), including bipolar disorder (8.9 *v.* 5.1%). Personality disorders are balanced (approximately 3% in both genders).

The average length of stay is statistically higher for men, with 5.55 days spent in ICU compared with 4.4 days for women (*p* < 0.0001). The Charlson score is also higher in men (4.1% of men with Charlson score >3 *v*. 2.5% of women). Women are more likely to enter a psychiatric ward 3 days after their discharge from ICU (*N* = 16 912; 38.5% of women) than men (*N* = 12 309; 32.4% of men).

### Comparison by fatal outcome

Following a first MSSA, 4519 patients died, representing 5.5% of the MSSA population (Table 2). MSSAs are significantly more lethal in men, resulting in death in 8.1% of cases *v*. 3.3% of women (*p* < 0.0001). On average, the deceased are older (54.69 ± 19.23 years) than the survivors (45.28 ± 17.41 years). A high case fatality rate is observed over 75 years of age, with 15% of deaths compared to 4.4% in the (25–55) age group. The proportion of fatal outcomes after an MSSA in patients admitted from psychiatry (11.4%) is higher than the same proportion in patients admitted from any other place (5.5%). The number of deaths to MSSA is stable throughout the study period (every year about 12–13% of the total).

**Table 2.** Demographic and clinical features of all MSSA patients by living *v.* deceased status

	Living following first MSSA *N* = 77 440 *N* (row percentage)	Died following first MSSA *N* = 4519 *N* (row percentage)	Total *N* = 81 959 *N* (column percentage)	*p*-Value^a^
Male	34 916 (91.9%)	3090 (8.1%)	38 006 (46.4%)	
Female	42 524 (96.7%)	1429 (3.3%)	43 953 (53.6%)	
Age (mean ± s.d.)	45.28 ± 17.41	54.69 ± 19.23	45.8 ± 17.64	**<0.0001**
Age in five classes				
[8 years; 15 years]	398 (96.6%)	14 (3.4%)	412 (0.5%)	**<0.0001**
[15 years; 25 years]	10 848 (97.6%)	272 (2.4%)	11 120 (13.6%)	
[25 years; 55 years]	44 111 (95.6%)	2039 (4.4%)	46 150 (56.3%)	
[55 years; 75 years]	17 242 (92.8%)	1341 (7.2%)	18 583 (22.7%)	
75+ years	4841 (85%)	853 (15%)	5694 (6.9%)	
Admission				
From emergency room	51 636 (95.2%)	2622 (4.8%)	54 258 (66.2%)	**<0.0001**
From a psychiatric unit	264 (88.6%)	34 (11.4%)	298 (0.4%)	
From home	23 456 (93.2%)	1718 (6.8%)	25 174 (30.7%)	
Other	2053 (93.5%)	142 (6.5%)	2195 (2.7%)	
Discharge				**<0.0001**
To home	37 652 (100%)	0	37 652 (45.9%)	
To psychiatric unit	25 177 (100%)	0	25 177 (30.7%)	
Other	14 611 (100%)	0	14 611 (17.8%)	
MSSA mechanism				
Drug poisoning (X60–64)	52 155 (98.1%)	1012 (1.9%)	53 167 (64.9%)	**<0.0001**
Other (X65–X84)	25 285 (87.8%)	3507 (12.2%)	28 792 (35.1%)	
Days in intensive care (mean ± s.d.)	7.13 ± 13.23	8.57 ± 20.31	7.21 ± 13.72	**<0.0001**
Charlson score				
0 (1-year mortality = 12%)	69 592 (94.8%)	3800 (5.2%)	73 392 (89.5%)	**<0.0001**
1–2 (1-year mortality = 26%)	5513 (92.8%)	425 (7.2%)	5938 (7.2%)	
3–4 (1-year mortality = 52%)	1509 (89.4%)	178 (10.6%)	1687 (2.1%)	
5+ (1-year mortality = 85%)	826 (87.7%)	116 (12.3%)	942 (1.1%)	
Alcohol SUD	19 754 (97.2%)	572 (2.8%)	20 326 (34.3%)	**<0.001**
Bipolar disorder	4118 (96.9%)	132 (3.1%)	4250 (7.2%)	**0.13**
Schizophrenia	2111 (96.6%)	74 (3.4%)	2185 (3.7%)	**0.74**
ICD 10 codes for mental and behavioural disorders^b^				
F01–F09	2291 (95.5%)	108 (4.5%)	2399 (4.1%)	**0.007**
F10–F19	26 593 (97.2%)	753 (2.8%)	27 346 (46.2%)	**<0.0001**
F20–F29	4368 (96.8%)	144 (3.2%)	4512 (7.6%)	0.22
F30–F39	31 624 (96.2%)	1263 (3.8%)	32 887 (55.6%)	**<0.009**
F40–F48	7773 (98%)	158 (2%)	7931 (13.4%)	**<0.0001**
F50–F59	657 (97.5%)	17 (2.5%)	674 (1.1%)	0.19
F60–F69	2025 (98.9%)	22 (1.1%)	2047 (3.5%)	**<0.0001**
F70–F79	126 (96.9%)	4 (3.1%)	130 (0.2%)	1
F80–F89	58 (98.3%)	1 (1.7%)	59 (0.1%)	_
F90–F98	822 (97.7%)	19 (2.3%)	841 (1.4%)	**0.047**
F99	697 (97.5%)	18 (2.5%)	715 (1.2%)	0.15
Psychiatric admission in the 3 days following discharge	29 221 (100%)	0	29 221 (35.7%)	

a *p*-Value for comparison between living *v.* deceased status, we used the Pearson *χ*^2^ tests for qualitative variables, and the Student *t*-test for quantitative variables.

b F00–F09: organic, including symptomatic, mental disorders; F10–F19: mental and behavioural disorders due to psychoactive substance use; F20–F29: schizophrenia, schizotypal and delusional disorders; F30–F39: mood (affective) disorders; F40–F48: neurotic, stress-related and somatoform disorders; F50–F59: behavioural syndromes associated with physiological disturbances and physical factors; F60–F69: disorders of adult personality and behaviour; F70–F79: mental retardation; F80–F89: disorders of psychological development; F90–F98: behavioural and emotional disorders with onset usually occurring in childhood and adolescence; F99: unspecified mental disorder.

Bold text indicates *p*-values ≤ 0.5.

DSP by drugs is less lethal (1.9% deaths) than any other method used in MSSAs (12.2% deaths). Among the deceased, the method chosen to attempt suicide is commonly violent. Hanging/strangulation is used in 38.5% of deaths but represents only 4.7% of all MSSAs, firearms are used in 9.8% of deaths and 1.6% of MSSAs. On the contrary, DSP accounts for 67.3% of MSSAs and 22.4% of deaths.

Mortality is particularly important in patients diagnosed with mood disorders or organic mental disorders (F3 and F0) when compared to any other diagnosis. On the contrary, diagnoses of SUD (F1), psychotic disorder (F2) or personality disorder (F6) are less often associated with a fatal outcome. Deceased patients have longer lengths of stay (8.57 ± 20.31 days) than survivors do (7.13 ± 13.23 days). In addition, the Charlson score is statistically higher in the decedents (15.9% with Charlson score >1 in the decedents *v*. 10.1% in the survivors).

### Discharge to a psychiatric service

One-third of surviving MSSA patients are discharged directly to a psychiatric hospital (*N* = 25 177, 32.5%), while almost half are discharged directly to their home (*N* = 37 652; 48.6%) (Table 3). These numbers are stable over the study period. Women are more likely to be discharged to psychiatry than men. They represent 58.5% of discharges to a psychiatric hospital, whereas they constitute 54.9% of survivors. The extreme age groups are less likely to be hospitalised in psychiatry after ICU, less than 25% in the youngest and oldest age groups (8–15 years and over 75 years) compared to more than 30% in the other age groups (25–75 years).

**Table 3.** Demographic and clinical features of patients discharged to a psychiatric service after their first MSSA (*n* = 77 440 patients)

	Discharge to a psychiatric unit: 0 *N* = 52 263 *N* (row percentage)	Discharge to a psychiatric unit: 1 *N* = 25 177 *N* (row percentage)	Total *N* = 77 440 *N* (column percentage)	*p*-Value^a^
Male	24 460 (70.1%)	10 456 (29.9%)	34 916 (45.1%)	**<0.0001**
Female	27 803 (65.4%)	14 721 (34.6%)	42 524 (54.9%)	
Age (mean ± s.d.)	45.13 ± 17.93	45.58 ± 16.27	45.28 ± 17.41	**0.0006**
Age in five classes				
[8 years; 15 years]	317 (79.6%)	81 (20.4%)	398 (0.5%)	**<0.0001**
[15 years; 25 years]	7735 (71.3%)	3113 (28.7%)	10 848 (14%)	
[25 years; 55 years]	29 390 (66.6%)	14 721 (33.4%)	44 111 (57%)	
[55 years;75 years]	11 099 (64.4%)	6143 (35.6%)	17 242 (22.3%)	
75+ years	3722 (76.9%)	1119 (23.1%)	4841 (6.3%)	
Admission				
From emergency room	34 111 (66.1%)	17 525 (33.9%)	51 636 (66.7%)	**<0.0001**
From a psychiatric unit	59 (22.3%)	205 (77.7%)	264 (0.3%)	
From home	16 528 (70.5%)	6928 (29.5%)	23 456 (30.3%)	
Other	1541 (75.1%)	512 (24.9%)	2053 (2.7%)	
Discharge				
To home	37 652 (100%)	0	37 652 (48.6%)	**<0.0001**
To psychiatric unit	0	25 177 (100%)	25 177 (32.5%)	
Other	14 611 (100%)	0	14 611 (18.9%)	
MSSA mechanism				
Drug poisoning (X60–64)	34 296 (65.8%)	17 859 (34.2%)	52 155 (67.3%)	**<0.0001**
Other (X65–X84)	17 967 (71.1%)	7318 (28.9%)	25 285 (32.7%)	
Days in intensive care (mean ± s.d.)	8.34 ± 15.17	4.61 ± 7.2	7.13 ± 13.23	**<0.0001**
Charlson score				
0 (1-year mortality = 12%)	46 460 (66.8%)	23 132 (33.2%)	69 592 (89.9%)	**<0.0001**
1–2 (1-year mortality = 26%)	3955 (71.7%)	1558 (28.3%)	5513 (7.1%)	
3–4 (1-year mortality = 52%)	1168 (77.4%)	341 (22.6%)	1509 (1.9%)	
5+ (1-year mortality = 85%)	680 (82.3%)	146 (17.7%)	826 (1.1%)	
Alcohol SUD	14 142 (71.6%)	5612 (28.4%)	19 754 (34.6%)	**<0.0001**
Bipolar disorder	2130 (51.7%)	1988 (48.3%)	4118 (7.2%)	**<0.0001**
Schizophrenia	1120 (53.1%)	991 (46.9%)	2111 (3.7%)	**<0.0001**
ICD 10 codes for mental and behavioural disorders^b^				
F01–F09	1735 (75.7%)	556 (24.3%)	2291 (4%)	**<0.0001**
F10–F19	18 954 (71.3%)	7639 (28.7%)	26 593 (46.6%)	**<0.0001**
F20–F29	2399 (54.9%)	1969 (45.1%)	4368 (7.7%)	**<0.0001**
F30–F39	19 781 (62.6%)	11 843 (37.4%)	31 624 (55.4%)	**<0.0001**
F40–F48	5456 (70.2%)	2317 (29.8%)	7773 (13.6%)	**<0.0001**
F50–F59	441 (67.1%)	216 (32.9%)	657 (1.2%)	**<0.0001**
F60–F69	1313 (64.8%)	712 (35.2%)	2025 (3.5%)	0.10
F70–F79	91 (72.2%)	35 (27.8%)	126 (0.2%)	0.18
F80–F89	36 (62.1%)	22 (37.9%)	58 (0.1%)	0.47
F90–F98	572 (69.6%)	250 (30.4%)	822 (1.4%)	0.06
F99	440 (63.1%)	257 (36.9%)	697 (1.2%)	0.06
Psychiatric admission in the 3 days following discharge	8093 (27.7%)	21 128 (72.3%)	29 221 (37.7%)	**<0.0001**

a *p*-Value for comparison according to the decision of discharge to a psychiatric unit or otherwise, we used the Pearson *χ*^2^ tests for qualitative variables and the Student *t*-test for quantitative variables.

b F00–F09: organic, including symptomatic, mental disorders; F10–F19: mental and behavioural disorders due to psychoactive substance use; F20–F29: schizophrenia, schizotypal and delusional disorders; F30–F39: mood (affective) disorders; F40–F48: neurotic, stress-related and somatoform disorders; F50–F59: behavioural syndromes associated with physiological disturbances and physical factors; F60–F69: disorders of adult personality and behaviour; F70–F79: mental retardation; F80–F89: disorders of psychological development; F90–F98: behavioural and emotional disorders with onset usually occurring in childhood and adolescence; F99: unspecified mental disorder.

Bold text indicates *p*-values ≤ 0.5.

MSSA survivors after DSP are more frequently hospitalised in psychiatry than those using different methods (34.2 *v*. 28.9%). Discharges to psychiatric hospitals after a violent MSSA, such as hanging (*N* = 925; 24%) or the use of a firearm (*N* = 167; 12.9%), are particularly infrequent.

Patients with psychotic disorder (*N* = 1969; 45.1%) or mood disorder (*N* = 11 843; 37.4%) are more often hospitalised in psychiatry, in contrast to those with SUD (*N* = 7639; 28.7%). Nearly three-quarters (*N* = 205; 77.7%) of psychiatric inpatients are re-admitted after intensive care in a psychiatric ward. On the other hand, about one-third of MSSA survivors coming from the emergency room (*N* = 17 525; 33.9%) or home (*N* = 6928; 29.5%) are subsequently admitted to a psychiatric hospital.

Patients who have fewer comorbidities are more often discharged to psychiatry (33.2% with Charlson score = 0 *v*. 17.7% with Charlson score > 5). Patients discharged to psychiatry wards also spend less time in intensive care than those discharged elsewhere (4.61 ± 7.2 *v*. 8.34 ± 15.17 days). Methods of attempting suicide are represented in Table 4.

**Table 4.** Demographic and clinical features of MSSA patients according to the main method used in the suicide attempt

	Intentional self-poisoning with drugs (X60–X64) *N* = 53 167 *N* (%)	Intentional self-poisoning with other substances (alcohol, solvents, pesticides, chemicals) (X65–X69) *N* = 7673 *N* (%)	Intentional self-harm by hanging, strangulation and suffocation (X70) *N* = 3857 *N* (%)	Intentional self-harm by drowning and submersion (X71) *N* = 256 *N* (%)	Intentional self-harm by firearm (X72–X74) *N* = 1297 *N* (%)	Intentional self-harm by smoke, fire and flames (X75–X77) *N* = 609 *N* (%)
Male	21 472 (56.5%)	4359 (11.5%)	3010 (7.9%)	102 (0.3%)	1196 (3.1%)	399 (1%)
Female	31 695 (72.1%)	3314 (7.5%)	847 (1.9%)	154 (0.4%)	101 (0.2%)	210 (0.5%)
Age in five classes						
[8 years; 15 years]	265 (64.3%)	30 (7.3%)	23 (5.6%)	1 (0.2%)	1 (0.2%)	2 (0.5%)
[15 years; 25 years]	7446 (67%)	875 (7.9%)	375 (3.4%)	12 (0.1%)	64 (0.6%)	56 (0.5%)
[25 years; 55 years]	29 926 (64.8%)	4471 (9.7%)	2452 (5.3%)	63 (0.1%)	490 (1.1%)	381 (0.8%)
[55 years; 75 years]	11 905 (64.1%)	1900 (10.2%)	769 (4.1%)	129 (0.7%)	462 (2.5%)	154 (0.8%)
75+ years	3625 (63.7%)	397 (7%)	238 (4.2%)	51 (0.9%)	280 (4.9%)	16 (0.3%)
Admission						
From emergency room	36 254 (66.8%)	5000 (9.2%)	2191 (4%)	161 (0.3%)	801 (1.5%)	217 (0.4%)
From a psychiatric unit	165 (55.4%)	20 (6.7%)	52 (17.4%)	2 (0.7%)	2 (0.7%)	4 (1.3%)
From home	15 425 (61.3%)	2432 (9.7%)	1527 (6.1%)	89 (0.4%)	445 (1.8%)	337 (1.3%)
Other	1306 (59.5%)	215 (9.8%)	86 (3.9%)	3 (0.1%)	48 (2.2%)	51 (2.3%)
Missing	17	6	1	1	1	0
Discharge						
To home	26 324 (69.9%)	4293 (11.4%)	674 (1.8%)	65 (0.2%)	324 (0.9%)	104 (0.3%)
To psychiatric unit	17 859 (70.9%)	1903 (7.6%)	925 (3.7%)	82 (0.3%)	167 (0.7%)	54 (0.2%)
Other	7972 (54.6%)	1183 (8.1%)	516 (3.5%)	44 (0.3%)	362 (2.5%)	314 (2.1%)
Death because of the attempt	1012 (22.4%)	294 (6.5%)	1742 (38.5%)	65 (1.4%)	444 (9.8%)	137 (3%)
Charlson score						
0 (1-year mortality = 12%)	47 569 (64.8%)	6788 (9.2%)	3589 (4.9%)	226 (0.3%)	1102 (1.5%)	542 (0.7%)
1–2 (1-year mortality = 26%)	3943 (66.4%)	584 (9.8%)	183 (3.1%)	21 (0.4%)	118 (2%)	51 (0.9%)
3–4 (1-year mortality = 52%)	1061 (62.9%)	185 (11%)	59 (3.5%)	5 (0.3%)	45 (2.7%)	13 (0.8%)
5+ (1-year mortality = 85%)	594 (63.1%)	116 (12.3%)	26 (2.8%)	4 (0.4%)	32 (3.4%)	3 (0.3%)
ICD 10 codes for mental and behavioural disorders^a^						
F01–F09	1178 (49.1%)	220 (9.2%)	113 (4.7%)	17 (0.7%)	80 (3.3%)	13 (0.5%)
F10–F19	17 690 (64.7%)	3678 (13.4%)	853 (3.1%)	42 (0.2%)	292 (1.1%)	139 (0.5%)
F20–F29	2600 (57.6%)	247 (5.5%)	115 (2.5%)	15 (0.3%)	34 (0.8%)	101 (2.2%)
F30–F39	22 376 (68%)	2419 (7.4%)	1429 (4.3%)	121 (0.4%)	503 (1.5%)	209 (0.6%)
F40–F48	5539 (69.8%)	578 (7.3%)	261 (3.3%)	30 (0.4%)	88 (1.1%)	63 (0.8%)
F50–F59	486 (72.1%)	38 (5.6%)	17 (2.5%)	0	4 (0.6%)	3 (0.4%)
F60–F69	1292 (63.1%)	101 (4.9%)	86 (4.2%)	6 (0.3%)	22 (1.1%)	28 (1.4%)
F70–F79	78 (60%)	11 (8.5%)	3 (2.3%)	1 (0.8%)	1 (0.8%)	3 (2.3%)
F80–F89	31 (52.5%)	3 (5.1%)	3 (5.1%)	1 (1.7%)	0	2 (3.4%)
F90–F98	516 (61.4%)	78 (9.3%)	30 (3.6%)	3 (0.4%)	17 (2%)	3 (0.4%)
F99	128 (17.9%)	14 (2%)	6 (0.8%)	1 (0.1%)	3 (0.4%)	2 (0.3%)
Alcohol use disorder	12 595 (62%)	3259 (16%)	646 (3.2%)	39 (0.2%)	238 (1.2%)	101 (0.5%)
Bipolar disorder	3054 (71.9%)	281 (6.6%)	86 (2%)	11 (0.3%)	35 (0.8%)	35 (0.8%)
Schizophrenia	1293 (59.2%)	103 (4.7%)	50 (2.3%)	5 (0.2%)	19 (0.9%)	42 (1.9%)
Missing	14 487	1998	1596	73	503	201

	Intentional self-harm by sharp or blunt object (X78–X79) *N* = 2308 *N* (%)	Intentional self-harm by jumping from a high place (X80) *N* = 2386 *N* (%)	Intentional self-harm by collision (X81–X82) *N* = 316 *N* (%)	Intentional self-harm by other specified or unspecified means (X83–X84) *N* = 10 090 *N* (%)	Total *N* = 81 959
Male	1462 (3.8%)	1214 (3.2%)	209 (0.5%)	4583 (12.1%)	38 006 (46.4%)
Female	846 (1.9%)	1172 (2.7%)	107 (0.2%)	5507 (12.5%)	43 953 (53.6%)
Age in five classes					
[8 years; 15 years]	16 (3.9%)	29 (7%)	0	45 (10.9%)	412 (0.5%)
[15 years; 25 years]	329 (3%)	508 (4.6%)	68 (0.6%)	1387 (12.5%)	11 120 (13.6%)
[25 years; 55 years]	1171 (2.5%)	1287 (2.8%)	181 (0.4%)	5728 (12.4%)	46 150 (56.3%)
[55 years; 75 years]	554 (3%)	390 (2.1%)	55 (0.3%)	2265 (12.2%)	18 583 (22.7%)
75+ years	238 (4.2%)	172 (3%)	12 (0.2%)	665 (11.7%)	5694 (6.9%)
Admission					
From emergency room	1551 (2.9%)	1392 (2.6%)	203 (0.4%)	6488 (12%)	54 258 (66.2%)
From a psychiatric unit	15 (5%)	6 (2%)	0	32 (10.7%)	298 (0.4%)
From home^a^	696 (2.8%)	873 (3.5%)	100 (0.4%)	3250 (12.9%)	25 174 (30.7%)
Other	44 (2%)	111 (5.1%)	13 (0.6%)	318 (14.5%)	2195 (2.7%)
Missing	2	4	0	2	34
Discharge					
To home^a^	892 (2.4%)	576 (1.5%)	98 (0.3%)	4302 (11.4%)	37 652 (45.9%)
To psychiatric unit	826 (3.3%)	298 (1.2%)	60 (0.2%)	3003 (11.9%)	25 177 (30.7%)
Other	510 (3.5%)	1300 (8.9%)	142 (1%)	2268 (15.5%)	14 611 (17.8%)
Death because of the attempt	80 (1.8%)	212 (4.7%)	16 (0.4%)	517 (11.4%)	4519 (5.5%)
Charlson score					
0 (1-year mortality = 12%)	2000 (2.7%)	2197 (3%)	299 (0.4%)	9080 (12.4%)	73 392 (89.5%)
1–2 (1-year mortality = 26%)	186 (3.1%)	132 (2.2%)	10 (0.2%)	710 (12%)	5938 (7.2%)
3–4 (1-year mortality = 52%)	79 (4.7%)	41 (2.4%)	6 (0.4%)	193 (11.4%)	1687 (2.1%)
5+ (1-year mortality = 85%)	43 (4.6%)	16 (1.7%)	1 (0.1%)	107 (11.4%)	942 (1.1%)
ICD 10 codes for mental and behavioural disorders^a^					
F01–F09	78 (3.3%)	170 (7.1%)	13 (0.5%)	517 (21.6%)	2399 (4.1%)
F10–F19	698 (2.6%)	725 (2.7%)	97 (0.4%)	3132 (11.5%)	27 346 (46.2%)
F20–F29	247 (5.5%)	454 (10.1%)	36 (0.8%)	663 (14.7%)	4512 (7.6%)
F30–F39	1006 (3.1%)	814 (2.5%)	124 (0.4%)	3886 (11.8%)	32 887 (55.6%)
F40–F48	241 (3%)	198 (2.5%)	30 (0.4%)	903 (11.4%)	7931 (13.4%)
F50–F59	20 (3%)	18 (2.7%)	0	88 (13.1%)	674 (1.1%)
F60–F69	114 (5.6%)	65 (3.2%)	12 (0.6%)	321 (15.7%)	2047 (3.5%)
F70–F79	5 (3.8%)	10 (7.7%)	1 (0.8%)	17 (13.1%)	130 (0.2%)
F80–F89	2 (3.4%)	6 (10.2%)	0	11 (18.6%)	59 (0.1%)
F90–F98	29 (3.4%)	30 (3.6%)	4 (0.5%)	131 (15.6%)	841 (1.4%)
F99	8 (1.1%)	17 (2.4%)	2 (0.3%)	534 (74.7%)	715 (1.2%)
Alcohol use disorder	492 (2.4%)	516 (2.5%)	70 (0.3%)	2370 (11.7%)	20 326 (34.3%)
Bipolar disorder	132 (3.1%)	134 (3.2%)	14 (0.3%)	468 (11%)	4250 (7.2%)
Schizophrenia	114 (5.2%)	248 (11.4%)	20 (0.9%)	291 (13.3%)	2185 (3.7%)
Missing	521	537	88	2779	22 783

a F00–F09: organic, including symptomatic, mental disorders; F10–F19: mental and behavioural disorders due to psychoactive substance use; F20–F29: schizophrenia, schizotypal and delusional disorders; F30–F39: mood (affective) disorders; F40–F48: neurotic, stress-related and somatoform disorders; F50–F59: behavioural syndromes associated with physiological disturbances and physical factors; F60–F69: disorders of adult personality and behaviour; F70–F79: mental retardation; F80–F89: disorders of psychological development; F90–F98: behavioural and emotional disorders with onset usually occurring in childhood and adolescence; F99: unspecified mental disorder.

### Incidence

We find an overall increase in the incidence of MSSAs over the study period, from 16.57 in 2012 to 17.75 in 2019 (cases per 1 00 000 person-years). This tendency was particularly marked in females aged 15–25 years with a 10-point increase (Table 5). In women, the incidence is consistently higher than in men, with a trend towards a narrowing of this gap. In 2012, the incidence is 17.42 in women and 15.66 in men. In 2019, 18.14 in women *v*. 17.33 in men.

**Table 5.** Incidence of the first MSSA per 100 000 person-years by age group and sex

	2012			2013			2014			2015			2016			2017			2018			2019		
	Male	Female	Both	Male	Female	Both	Male	Female	Both	Male	Female	Both	Male	Female	Both	Male	Female	Both	Male	Female	Both	Male	Female	Both
Age in five classes																								
[8 years; 15 years]	0.31	1.63	0.95	0.41	1.19	0.79	0.44	1.68	1.05	0.41	1.24	0.82	0.33	1.47	0.89	0.40	1.15	0.77	0.43	1.57	0.99	0.27	1.50	0.87
[15 years; 25 years]	13.07	17.30	15.16	12.14	18.55	15.29	15.74	20.58	18.12	13.28	19.49	16.33	13.48	19.00	16.19	13.54	21.73	17.55	15.74	25.19	20.37	16.51	28.13	22.21
[25 years; 55 years]	22.29	22.55	22.42	22.08	21.57	21.82	24.41	24.65	24.53	22.93	21.71	22.31	22.23	21.06	21.63	23.31	21.69	22.49	24.27	22.06	23.15	24.34	21.79	23.05
[55 years; 75 years]	12.42	17.51	15.08	12.87	16.65	14.85	13.85	18.03	16.04	14.12	17.66	15.97	12.81	17.03	15.02	15.11	18.17	16.72	14.54	17.24	15.96	15.10	16.85	16.02
75+ years	12.05	11.08	11.44	11.65	9.29	10.17	12.68	11.35	11.85	12.29	11.47	11.78	12.77	11.91	12.24	12.29	11.24	11.65	13.05	12.04	12.43	11.25	11.25	11.25
All ages^a^	15.66	17.42	16.57	15.48	16.68	16.10	17.30	18.84	18.10	16.31	17.33	16.84	15.68	16.90	16.31	16.67	17.67	17.19	17.25	18.11	17.70	17.33	18.14	17.75

Incidence rates were estimated using the exhaustive number of first MSSA in France by age, sex and year as the numerator and the French population size by age, sex and year as the denominator. The population size is based on an annual national census performed by the French ‘National Institute of Statistics and Economic Studies’ (INSEE) and are directly available on the Institute's website (https://www.insee.fr/fr/statistiques/1913143?sommaire = 1912926).

a Incidence in the population over 8 years old.

## Discussion

To our knowledge, this is the largest study to date focused on MSSA. It provides an 8-year coverage of first MSSAs in the entire French population aged 8 or more and allows us to explore an important link of the ideation to action to death continuum. The resulting sample (*n* = 81 859) comprised roughly equal percentages of men and women, with a slight female majority. The most common method of attempting suicide in both sexes was DSP, although violent methods were over-represented in men and associated with higher mortality and comorbidity. These results are in accordance with the literature (Beautrais, 2001, 2003b; Giner *et al*., 2014; Kim *et al*., 2020). More surprisingly, almost one in two MSSA went directly home after intensive care. These cases concern mainly men with psychiatric comorbidity and using violent methods. Precisely those MSSA survivors that are most at risk for suicide are the least hospitalised. For instance, one in four MSSA were discharged home after using violent methods such as firearms or jumping from a high place. Concerning psychopathology, we found an almost equal prevalence of mood disorders (55.6%) and SUDs (46.2%) in the sample. They were by large the most common psychiatric disorders and showed a clear gender pattern, women were more often diagnosed with mood disorders and men with SUDs.

One in 20 MSSA attempters died in our sample, which represents more than fivefold the death rate by any SA leading to hospitalisation in France between 2004 and 2011 (Chee and Jezewski-Serra, 2014). Similar to suicide victims (Värnik *et al*., 2008; Cibis *et al*., 2012; Bachmann, 2018), the profile of MSSA attempters who die corresponds to a man over 65 years that used hanging as a method. MSSA attempters are older compared to other suicide attempters (Horesh *et al*., 2012; Giner *et al*., 2014; Kim *et al*., 2020), but younger than suicide victims (Beautrais, 2001).

The ‘gender paradox’ in suicide posits that women make more attempts but men die more often (Canetto and Sakinofsky, 1998; Schrijvers *et al*., 2012). In our study, men represent 68.4% of deaths, spend more time in intensive care and have more somatic conditions than women. These differences could be explained by the choice of more lethal methods in men (Beautrais, 2003b) but even comparing similar methods men have higher mortality rates in our sample. However, the risk of suicide among women seems to be particularly high after an MSSA (Beautrais, 2004).

Two out of three MSSAs used DSP as a method for suicide. Similar results were found in previous studies with intoxications accounting for 57–79% of MSSAs (Beautrais, 2003b; Horesh *et al*., 2012; Sun *et al*., 2015; Kim *et al*., 2020). SA methods in our sample are thus closer to those of low-lethality SAs than to suicides if we compare with previous studies: (i) 81.7% of suicides used a highly lethal method against 17.6% in MSSAs (Beautrais, 2003b), (ii) DSP accounts for approximately two out of three SAs not requiring intensive care (Cibis *et al*., 2012), but (iii) DSP accounts only for 12.7% of suicides in Europe (Värnik *et al*., 2008).

One in 10 patients with MSSA (10.5%) had multiple somatic comorbidities. Indeed, physical illnesses can precipitate MSSAs (Kim *et al*., 2020). Patients with somatic pathology are 2–3 times more likely to die by suicide compared to the general population in Taiwan (Cheng *et al*., 2000), even more so in case of multiple co-morbidities or social isolation (Ahmedani *et al*., 2017; Kennedy and Garmon-Jones, 2017). In the same vein, intensive care survivors have more risk of suicide after discharge than other inpatients (Fernando *et al*., 2021). According to a national population study, the interaction of psychiatric and somatic illnesses increases the risk of suicide (Qin *et al*., 2014), and this trend seems to be particularly significant in elderly men (Blasco-Fontecilla *et al*., 2010). Somatic conditions often precipitate suicidal acts in the elderly, which present an elevated fatality rate post-MSSA. Physical pain interacts with psychological pain, cognitive impairments and loneliness facilitating more severe attempts in this age group (Conejero *et al*., 2018). It should also be noted that the physical consequences of an MSSA may increase suicide risk and can hinder psychiatric care, especially after a violent SA (Persett *et al*., 2018). Overall mortality is sharply increased after violent self-harm, especially during the first year, and also among men and physically vulnerable persons (Chen *et al*., 2011; Bergen *et al*., 2012; Stenbacka and Jokinen, 2015; Goldman-Mellor *et al*., 2019; Vuagnat *et al*., 2019).

High levels of psychopathology increase the susceptibility to make an MSSA (Beautrais, 2003b). The diagnostic profile of MSSA patients (mood disorders, SUDs) in our study is similar to previous reports by Beautrais's group, which found high rates of current and lifetime mental disorders (around 90%), and previous SAs (23.6–52.7%) (Beautrais *et al*., 1996, 1998; Beautrais, 2001). Post-traumatic stress disorder (PTSD) was also more prevalent among MSSA survivors (Lopez-Castroman *et al*., 2015). Consistently with previous studies (Beautrais *et al*., 1999; Gvion and Levi-Belz, 2018), SUDs and particularly AUDs were very frequent in our sample, one in three MSSA survivors (31.1%) had an AUD in the previous month (Beautrais *et al*., 1999). Alcohol dependence more than doubles the risk of making an MSSA compared to the general population (Conner *et al*., 2003a), and drinking within 3 h of an SA causes a sixfold increase in the risk of MSSA (Powell *et al*., 2001). In a prior study, the comorbidity of a mood disorder with alcohol dependence conveyed lower odds of an MSSA compared to a mood disorder alone (OR = 6 *v*. 17), but alcohol-dependent patients making MSSAs were more likely to have a mood disorder (Conner *et al*., 2003a, 2003b). Other SUDs, such as cannabis or tobacco dependence, have been strongly associated with MSSAs (Beautrais *et al*., 1999; Lopez-Castroman *et al*., 2016).

The psychiatric profiles of suicide victims and MSSAs present strong similarities. Mood disorders, SUDs and anxiety disorders are very frequent in both groups compared to the general population (Beautrais, 2001; Arsenault-Lapierre *et al*., 2004). Nevertheless, suicide victims appear to be several times more likely to present non-affective psychoses compared to MSSA survivors that in turn would be more likely to present anxiety disorders and mood disorders (Beautrais, 2001, 2003b). Compared with SAs not requiring critical care, psychotic disorders, bipolar disorder, eating disorders, SUDs and SA recidivism are overrepresented in MSSAs (Giner *et al*., 2014; Kim *et al*., 2020).

In our study, only 32.5% of MSSA survivors were subsequently admitted in psychiatric hospitals. Male sex, long ICU stays and advanced age decreased the likelihood of admission in psychiatry after critical care according to our results. Between 2004 and 2011, 20% of all SAs hospitalised in French hospitals (MCO) were subsequently admitted in psychiatry (Chee and Jezewski-Serra, 2014). These low rates are partly explained by admissions in psychiatric units within general hospitals, which are not differentiated in our study. In France, these units are mostly conceived to provide emergency psychiatric care in academic hospitals, and despite an increase in recent years they are still uncommon and most psychiatric care takes place in psychiatric clinics or hospitals (public or private). Besides, stays in psychiatric emergency units are short and most patients in our study were discharged to their homes (48.6%).

Given the low rate of psychiatric hospitalisations in our study, improving care pathways for MSSA survivors seems essential. Any intervention targeting this gap should focus on high comorbidity (especially between SUDs and mood disorders) and the male sex. Co-morbid conditions may increase attrition (Lamers *et al*., 2012) and decrease the efficacy of treatments. For instance, the response to antidepressants is modest in AUDs with comorbid depression (Agabio *et al*., 2018) although it can be enhanced with cognitive behavioural therapy (Moak *et al*., 2003). Concerning gender, men have less knowledge and more stigmatizing attitudes about suicide than women do (Batterham *et al*., 2013), which may explain their tendency to seek help less often (Calear *et al*., 2014). The 2-year period following discharge after an SA is at high risk for suicide (Tejedor *et al*., 1999; Parra-Uribe *et al*., 2017). Continuous psychiatric and somatic outpatient care is very important. A 7-day follow-up after discharge from a psychiatric hospital was associated with an important decrease in suicide rates in the 3-month period following discharge (While *et al*., 2012). Specific training or sensibilisation of non-psychiatric teams about the risk of suicide and its management is important. Less than 70% of emergency physicians know how to assess the risk of suicide (Betz *et al*., 2013).

Non-psychiatric medical departments are not well-adapted to the management of suicide attempters (less communication, untrained teams) (Cheng *et al*., 2009; Betz *et al*., 2013; Chen *et al*., 2016). Patients admitted for self-harm to non-psychiatric departments are more likely to die by suicide in the following year than those admitted to psychiatric units (Vuagnat *et al*., 2019). Consultation-liaison psychiatry (CLP) should identify and manage these cases to reinforce continuous care (Stein *et al*., 2020), but the accessibility to CLP varies widely among hospitals (Olfson *et al*., 2014; Wood and Wand, 2014; Wand *et al*., 2016). According to one study, when CLP intervenes only 29.2% of violent or serious attempts are discharged (Cooper-Kazaz, 2013).

The incidence rate in our study is lower (45.7 per 1 00 000 person-years) compared to a Chinese study based on the 2009–2011 public health surveillance systems of three counties. However, their incidence rate included suicide deaths happening before arrival at the hospital (Sun *et al*., 2015). The incidence for all SAs (MSSAs included) was estimated to be 148.8 per 1 00 000 person-years in a US study (Kuo *et al*., 2001).

### Strengths and limitations of our study

The main limitation of this study is that it is based on administrative data, ICD-10 codes are mainly used for billing purposes by physicians in the hospital ward. They might vary according to the practitioner or the institution. Also, short stays, especially in services other than intensive care, may raise questions about the severity of the suicidal act, and important information such as suicidal intent or records of previous suicidality is not available in the database. Finally, the 2-year criterion we used to define a first MSSA is based on the diagnostic category of suicidal behaviour disorder, as defined in the Diagnostic and Statistical Manual of Mental Disorders (DSM5), but it may not completely reflect true incidence. However, PMSI data provide exhaustive and updated information on all health establishments at the national level for almost 10 years. This information completes the profile of MSSA attempters outlined by clinical studies and confirms the epidemiological importance of MSSA, as well as the limitations of current approaches to ensure that survivors access psychiatric care.

## Conclusions

MSSA survivors use more violent methods and have more serious somatic and psychiatric pathologies than low-lethality attempters. They are also at high risk of subsequent somatic complications or suicide. Very often they are discharged home instead of receiving inpatient psychiatric care, particularly those that are most at risk: men using violent methods or presenting comorbidities. The incidence of MSSAs is high, similar to that of suicides, and it seems essential to improve care pathways for MSSA survivors. This study needs to be extended with prospective data in order to better understand the consequences of MSSA and adapt care management.

## Data Availability

The data are not publicly available but will be provided upon request with the permission of the French Ministry of Health.

## References

[ref2] Agabio R, Trogu E and Pani PP (2018) Antidepressants for the treatment of people with co-occurring depression and alcohol dependence. The Cochrane Database of Systematic Reviews 4, CD008581. 29688573 10.1002/14651858.CD008581.pub2 PMC6494437

[ref3] Ahmedani BK, Peterson EL, Hu Y, Rossom RC, Lynch F, Lu CY, Waitzfelder BE, Owen-Smith AA, Hubley S, Prabhakar D, Williams LK, Zeld N, Mutter E, Beck A, Tolsma D and Simon GE (2017) Major physical health conditions and risk of suicide. American Journal of Preventive Medicine 53, 308–315. 28619532 10.1016/j.amepre.2017.04.001 PMC5598765

[ref4] Arsenault-Lapierre G, Kim C and Turecki G (2004) Psychiatric diagnoses in 3275 suicides: a meta-analysis. BMC Psychiatry 4, 37. 15527502 10.1186/1471-244X-4-37 PMC534107

[ref5] Bachmann S (2018) Epidemiology of suicide and the psychiatric perspective. International Journal of Environmental Research and Public Health 15, 1425. 10.3390/ijerph15071425 PMC6068947 29986446

[ref6] Batterham PJ, Calear AL and Christensen H (2013) Correlates of suicide stigma and suicide literacy in the community. Suicide & Life-Threatening Behavior 43, 406–417. 23556504 10.1111/sltb.12026

[ref7] Beautrais AL (2001) Suicides and serious suicide attempts: two populations or one? Psychological Medicine 31, 837–845. 11459381 10.1017/s0033291701003889

[ref8] Beautrais AL (2003a) Subsequent mortality in medically serious suicide attempts: a 5 year follow-up. The Australian and New Zealand Journal of Psychiatry 37, 595–599. 14511088 10.1046/j.1440-1614.2003.01236.x

[ref9] Beautrais AL (2003b) Suicide and serious suicide attempts in youth: a multiple-group comparison study. The American Journal of Psychiatry 160, 1093–1099. 12777267 10.1176/appi.ajp.160.6.1093

[ref10] Beautrais AL (2004) Further suicidal behavior among medically serious suicide attempters. Suicide & Life-Threatening Behavior 34, 1–11. 15106883 10.1521/suli.34.1.1.27772

[ref11] Beautrais AL, Joyce PR, Mulder RT, Fergusson DM, Deavoll BJ and Nightingale SK (1996) Prevalence and comorbidity of mental disorders in persons making serious suicide attempts: a case-control study. The American Journal of Psychiatry 153, 1009–1014. 8678168 10.1176/ajp.153.8.1009

[ref12] Beautrais AL, Joyce PR and Mulder RT (1998) Psychiatric illness in a New Zealand sample of young people making serious suicide attempts. The New Zealand Medical Journal 111, 44–48. 9539914

[ref13] Beautrais AL, Joyce PR and Mulder RT (1999) Cannabis abuse and serious suicide attempts. Addiction (Abingdon, England) 94, 1155–1164. 10615730 10.1046/j.1360-0443.1999.94811555.x

[ref14] Bergen H, Hawton K, Waters K, Ness J, Cooper J, Steeg S and Kapur N (2012) Premature death after self-harm: a multicentre cohort study. Lancet (London, England) 380, 1568–1574. 22995670 10.1016/S0140-6736(12)61141-6

[ref15] Betz ME, Sullivan AF, Manton AP, Espinola JA, Miller I, Camargo CA, Boudreaux and ED and ED-SAFE Investigators (2013) Knowledge, attitudes, and practices of emergency department providers in the care of suicidal patients. Depression and Anxiety 30, 1005–1012. 23426881 10.1002/da.22071 PMC4350671

[ref16] Blasco-Fontecilla H, Baca-Garcia E, Duberstein P, Perez-Rodriguez MM, Dervic K, Saiz-Ruiz J, Courtet P, de Leon J and Oquendo MA (2010) An exploratory study of the relationship between diverse life events and specific personality disorders in a sample of suicide attempters. Journal of Personality Disorders 24, 773–784. 21158599 10.1521/pedi.2010.24.6.773 PMC3057651

[ref17] Calear AL, Batterham PJ and Christensen H (2014) Predictors of help-seeking for suicidal ideation in the community: risks and opportunities for public suicide prevention campaigns. Psychiatry Research 219, 525–530. 25048756 10.1016/j.psychres.2014.06.027

[ref18] Canetto SS and Sakinofsky I (1998) The gender paradox in suicide. Suicide & Life-Threatening Behavior 28, 1–23. 9560163

[ref19] Charlson ME, Pompei P, Ales KL and MacKenzie CR (1987) A new method of classifying prognostic comorbidity in longitudinal studies: development and validation. Journal of Chronic Diseases 40, 373–383. 3558716 10.1016/0021-9681(87)90171-8

[ref20] Chee CC and Jezewski-Serra D (2014) Hospitalisations et recours aux urgences pour tentative de suicide en France métropolitaine à partir du PMSI-MCO 2004–2011 et d'Oscour® 2007–2011. Available at https://www.f2rsmpsy.fr/544-hospitalisations-recours-aux-urgences-pour-tentative-suicide-france-metropolitaine-partir-pmsi-mco-2004-2011-droscou.

[ref21] Chen VCH, Tan HKL, Chen C-Y, Chen THH, Liao L-R, Lee CTC, Dewey M, Stewart R, Prince M and Cheng ATA (2011) Mortality and suicide after self-harm: community cohort study in Taiwan. The British Journal of Psychiatry: The Journal of Mental Science 198, 31–36. 21200074 10.1192/bjp.bp.110.080952

[ref22] Chen KY, Evans R and Larkins S (2016) Why are hospital doctors not referring to consultation-liaison psychiatry? A systemic review. BMC Psychiatry 16, 390. 27829386 10.1186/s12888-016-1100-6 PMC5103418

[ref23] Cheng AT, Chen TH, Chen CC and Jenkins R (2000) Psychosocial and psychiatric risk factors for suicide. Case-control psychological autopsy study. The British Journal of Psychiatry: The Journal of Mental Science 177, 360–365. 11116779 10.1192/bjp.177.4.360

[ref24] Cheng I-C, Hu F-C and Tseng M-CM (2009) Inpatient suicide in a general hospital. General Hospital Psychiatry 31, 110–115. 19269530 10.1016/j.genhosppsych.2008.12.008

[ref25] Cibis A, Mergl R, Bramesfeld A, Althaus D, Niklewski G, Schmidtke A and Hegerl U (2012) Preference of lethal methods is not the only cause for higher suicide rates in males. Journal of Affective Disorders 136, 9–16. 21937122 10.1016/j.jad.2011.08.032

[ref26] Clark DC and Horton-Deutsch SL (1992) Assessment in absentia: the value of the psychological autopsy method for studying antecedents of suicide and predicting future suicides. In Maris RW, Berman AL, Maltsberger JT and Yufit RI (eds), Assessment and Prediction of Suicide. New York, NY, USA: Guilford Press, pp. 144–182.

[ref27] Conejero I, Olié E, Courtet P and Calati R (2018) Suicide in older adults: current perspectives. Clinical Interventions in Aging 13, 691–699. 29719381 10.2147/CIA.S130670 PMC5916258

[ref28] Conner KR, Beautrais AL and Conwell Y (2003a) Moderators of the relationship between alcohol dependence and suicide and medically serious suicide attempts: analyses of Canterbury Suicide Project data. Alcoholism Clinical and Experimental Research 27, 1156–1161. 12878922 10.1097/01.ALC.0000075820.65197.FD

[ref29] Conner KR, Beautrais AL and Conwell Y (2003b) Risk factors for suicide and medically serious suicide attempts among alcoholics: analyses of Canterbury Suicide Project data. Journal of Studies on Alcohol 64, 551–554. 12921197 10.15288/jsa.2003.64.551

[ref30] Cooper-Kazaz R (2013) Psychiatric consultation of all suicide-attempt patients during a one year period in a tertiary hospital. The Israel Medical Association Journal: IMAJ 15, 424–429. 24079063

[ref31] Fernando SM, Qureshi D, Sood MM, Pugliese M, Talarico R, Myran DT, Herridge MS, Needham DM, Rochwerg B, Cook DJ, Wunsch H, Fowler RA, Scales DC, Bienvenu OJ, Rowan KM, Kisilewicz M, Thompson LH, Tanuseputro P and Kyeremanteng K (2021) Suicide and self-harm in adult survivors of critical illness: population based cohort study. The BMJ 373, n973. 33952509 10.1136/bmj.n973 PMC8097311

[ref32] Giner L, Jaussent I, Olié E, Béziat S, Guillaume S, Baca-Garcia E, Lopez-Castroman J and Courtet P (2014) Violent and serious suicide attempters: one step closer to suicide? The Journal of Clinical Psychiatry 75, e191–e197. 24717390 10.4088/JCP.13m08524

[ref33] Goldman-Mellor S, Olfson M, Lidon-Moyano C and Schoenbaum M (2019) Association of suicide and other mortality with emergency department presentation. JAMA Network Open 2, e1917571. 31834399 10.1001/jamanetworkopen.2019.17571 PMC6991205

[ref34] Gvion Y and Levi-Belz Y (2018) Serious suicide attempts: systematic review of psychological risk factors. Frontiers in Psychiatry 9, 56. 29563886 10.3389/fpsyt.2018.00056 PMC5845877

[ref35] Han B, Kott PS, Hughes A, McKeon R, Blanco C and Compton WM (2016) Estimating the rates of deaths by suicide among adults who attempt suicide in the United States. Journal of Psychiatric Research 77, 125–133. 27032110 10.1016/j.jpsychires.2016.03.002

[ref36] Hawton K (2001) Studying survivors of nearly lethal suicide attempts: an important strategy in suicide research. Suicide & Life-Threatening Behavior 32, 76–84. 11924699 10.1521/suli.32.1.5.76.24215

[ref37] Hawton K and van Heeringen K (2009) Suicide. Lancet (London, England) 373, 1372–1381. 19376453 10.1016/S0140-6736(09)60372-X

[ref38] Hawton K, Appleby L, Platt S, Foster T, Cooper J, Malmberg A and Simkin S (1998) The psychological autopsy approach to studying suicide: a review of methodological issues. Journal of Affective Disorders 50, 269–276. 9858086 10.1016/s0165-0327(98)00033-0

[ref39] Hawton K, Sutton L, Haw C, Sinclair J and Harriss L (2005) Suicide and attempted suicide in bipolar disorder: a systematic review of risk factors. The Journal of Clinical Psychiatry 66, 693–704. 15960561 10.4088/jcp.v66n0604

[ref40] Hawton K, Bergen H, Cooper J, Turnbull P, Waters K, Ness J and Kapur N (2015) Suicide following self-harm: findings from the multicentre study of self-harm in England, 2000–2012. Journal of Affective Disorders 175, 147–151. 25617686 10.1016/j.jad.2014.12.062

[ref41] Horesh N, Levi Y and Apter A (2012) Medically serious versus non-serious suicide attempts: relationships of lethality and intent to clinical and interpersonal characteristics. Journal of Affective Disorders 136, 286–293. 22197510 10.1016/j.jad.2011.11.035

[ref42] Kennedy P and Garmon-Jones L (2017) Self-harm and suicide before and after spinal cord injury: a systematic review. Spinal Cord 55, 2–7. 27670807 10.1038/sc.2016.135

[ref43] Kessler RC, Berglund P, Borges G, Nock M and Wang PS (2005) Trends in suicide ideation, plans, gestures, and attempts in the United States, 1990–1992 to 2001–2003. JAMA 293, 2487–2495. 15914749 10.1001/jama.293.20.2487

[ref44] Kim S, Choi KH, Lee K-S, Kim D-J, Hong S-C, Lee H-K, Kweon Y-S, Lee CT and Lee K-U (2020) Risk factors for serious suicide attempts with high medical severity. Suicide & Life-Threatening Behavior 50, 408–421. 31642549 10.1111/sltb.12597

[ref45] Kuo WH, Gallo JJ and Tien AY (2001) Incidence of suicide ideation and attempts in adults: the 13-year follow-up of a community sample in Baltimore, Maryland. Psychological Medicine 31, 1181–1191. 11681544 10.1017/s0033291701004482

[ref46] Lamers F, Hoogendoorn AW, Smit JH, van Dyck R, Zitman FG, Nolen WA and Penninx BW (2012) Sociodemographic and psychiatric determinants of attrition in the Netherlands Study of Depression and Anxiety (NESDA). Comprehensive Psychiatry 53, 63–70. 21397218 10.1016/j.comppsych.2011.01.011

[ref47] Levi-Belz Y and Beautrais A (2016) Serious suicide attempts: toward an integration of terms and definitions. Crisis: The Journal of Crisis Intervention and Suicide Prevention 37, 299–309. 10.1027/0227-5910/a000386 27245812

[ref48] Lopez-Castroman J, Jaussent I, Beziat S, Guillaume S, Baca-Garcia E, Olié E and Courtet P (2015) Posttraumatic stress disorder following childhood abuse increases the severity of suicide attempts. Journal of Affective Disorders 170, 7–14. 25217758 10.1016/j.jad.2014.08.010

[ref49] Lopez-Castroman J, Cerrato L, Beziat S, Jaussent I, Guillaume S and Courtet P (2016) Heavy tobacco dependence in suicide attempters making recurrent and medically serious attempts. Drug and Alcohol Dependence 160, 177–182. 26832932 10.1016/j.drugalcdep.2016.01.004

[ref50] Moak DH, Anton RF, Latham PK, Voronin KE, Waid RL and Durazo-Arvizu R (2003) Sertraline and cognitive behavioral therapy for depressed alcoholics: results of a placebo-controlled trial. Journal of Clinical Psychopharmacology 23, 553–562. 14624185 10.1097/01.jcp.0000095346.32154.41

[ref51] Mościcki EK, O'Carroll P, Rae DS, Locke BZ, Roy A and Regier DA (1988) Suicide attempts in the epidemiologic catchment area study. The Yale Journal of Biology and Medicine 61, 259–268. 3262956 PMC2590450

[ref52] Olfson M, Marcus SC and Bridge JA (2014) Focusing suicide prevention on periods of high risk. JAMA 311, 1107–1108. 24515285 10.1001/jama.2014.501

[ref1] **Papon S, Beaumel C** (2020) Bilan démographique 2019. *Insee Première* **1789**. Available at https://www.insee.fr/fr/statistiques?collection=116.

[ref53] Parra-Uribe I, Blasco-Fontecilla H, Garcia-Parés G, Martínez-Naval L, Valero-Coppin O, Cebrià-Meca A, Oquendo MA and Palao-Vidal D (2017) Risk of re-attempts and suicide death after a suicide attempt: a survival analysis. BMC Psychiatry 17, 163. 28472923 10.1186/s12888-017-1317-z PMC5415954

[ref54] Persett PS, Grimholt TK, Ekeberg O, Jacobsen D and Myhren H (2018) Patients admitted to hospital after suicide attempt with violent methods compared to patients with deliberate self-poisoning – a study of background variables, somatic and psychiatric health and suicidal behavior. BMC Psychiatry 18, 21. 29368645 10.1186/s12888-018-1602-5 PMC5784599

[ref55] Posner K, Oquendo MA, Gould M, Stanley B and Davies M (2007) Columbia Classification Algorithm of Suicide Assessment (C-CASA): classification of suicidal events in the FDA's pediatric suicidal risk analysis of antidepressants. The American Journal of Psychiatry 164, 1035–1043. 17606655 10.1176/appi.ajp.164.7.1035 PMC3804920

[ref56] Powell KE, Kresnow MJ, Mercy JA, Potter LB, Swann AC, Frankowski RF, Lee RK and Bayer TL (2001) Alcohol consumption and nearly lethal suicide attempts. Suicide & Life-Threatening Behavior 32, 30–41. 11924693 10.1521/suli.32.1.5.30.24208

[ref57] Probert-Lindström S, Berge J, Westrin Å, Öjehagen A and Skogman Pavulans K (2020) Long-term risk factors for suicide in suicide attempters examined at a medical emergency in patient unit: results from a 32-year follow-up study. BMJ Open 10, e038794. 10.1136/bmjopen-2020-038794 PMC7783608 33130567

[ref58] Qin P, Hawton K, Mortensen PB and Webb R (2014) Combined effects of physical illness and comorbid psychiatric disorder on risk of suicide in a national population study. The British Journal of Psychiatry: The Journal of Mental Science 204, 430–435. 24578445 10.1192/bjp.bp.113.128785

[ref59] Schrijvers DL, Bollen J and Sabbe BGC (2012) The gender paradox in suicidal behavior and its impact on the suicidal process. Journal of Affective Disorders 138, 19–26. 21529962 10.1016/j.jad.2011.03.050

[ref60] Stein B, Müller MM, Meyer LK and Söllner W, CL Guidelines Working Group (2020) Psychiatric and psychosomatic consultation-liaison services in general hospitals: a systematic review and meta-analysis of effects on symptoms of depression and anxiety. Psychotherapy and Psychosomatics 89, 6–16. 31639791 10.1159/000503177

[ref61] Stenbacka M and Jokinen J (2015) Violent and non-violent methods of attempted and completed suicide in Swedish young men: the role of early risk factors. BMC Psychiatry 15, 196. 26271258 10.1186/s12888-015-0570-2 PMC4536779

[ref62] Sun J, Guo X, Zhang J, Wang M, Jia C and Xu A (2015) Incidence and fatality of serious suicide attempts in a predominantly rural population in Shandong, China: a public health surveillance study. BMJ Open 5, e006762. 10.1136/bmjopen-2014-006762 PMC4325129 25673439

[ref63] Tejedor MC, Díaz A, Castillón JJ and Pericay JM (1999) Attempted suicide: repetition and survival – findings of a follow-up study. Acta Psychiatrica Scandinavica 100, 205–211. 10493087 10.1111/j.1600-0447.1999.tb10847.x

[ref64] Värnik A, Kõlves K, van der Feltz-Cornelis CM, Marusic A, Oskarsson H, Palmer A, Reisch T, Scheerder G, Arensman E, Aromaa E, Giupponi G, Gusmäo R, Maxwell M, Pull C, Szekely A, Sola VP and Hegerl U (2008) Suicide methods in Europe: a gender-specific analysis of countries participating in the ‘European Alliance Against Depression’. Journal of Epidemiology and Community Health 62, 545–551. 18477754 10.1136/jech.2007.065391 PMC2569832

[ref65] Vuagnat A, Jollant F, Abbar M, Hawton K and Quantin C (2019) Recurrence and mortality 1 year after hospital admission for non-fatal self-harm: a nationwide population-based study. Epidemiology and Psychiatric Sciences 29, e20. 30773154 10.1017/S2045796019000039 PMC8061131

[ref66] Wand APF, Wood R, Macfarlane MD and Hunt GE (2016) Comparison of consultation-liaison psychiatry services for inner-city, district or regional general hospitals using a common tool: does one size fit all? Journal of Psychosomatic Research 84, 13–21. 27095154 10.1016/j.jpsychores.2016.03.007

[ref67] While D, Bickley H, Roscoe A, Windfuhr K, Rahman S, Shaw J, Appleby L and Kapur N (2012) Implementation of mental health service recommendations in England and Wales and suicide rates, 1997–2006: a cross-sectional and before-and-after observational study. Lancet (London, England) 379, 1005–1012. 22305767 10.1016/S0140-6736(11)61712-1

[ref68] Wood R and Wand APF (2014) The effectiveness of consultation-liaison psychiatry in the general hospital setting: a systematic review. Journal of Psychosomatic Research 76, 175–192. 24529036 10.1016/j.jpsychores.2014.01.002

